# Atypical Anxiety-Related Amygdala Reactivity and Functional Connectivity in Sant Mat Meditation

**DOI:** 10.3389/fnbeh.2018.00298

**Published:** 2018-12-04

**Authors:** Chenyi Chen, Yu-Chun Chen, Kuan-Ling Chen, Yawei Cheng

**Affiliations:** ^1^Department of Physical Medicine & Rehabilitation, National Yang-Ming University Hospital, Yilan, Taiwan; ^2^Graduate Institute of Injury Prevention and Control, College of Public Health, Taipei Medical University, Taipei, Taiwan; ^3^Institute of Humanities in Medicine, Taipei Medical University, Taipei, Taiwan; ^4^Research Center of Brain and Consciousness, Shuang Ho Hospital, Taipei Medical University, Taipei, Taiwan; ^5^Institute of Neuroscience and Brain Research Center, National Yang-Ming University, Taipei, Taiwan; ^6^Department of Research and Education, Taipei City Hospital, Taipei, Taiwan

**Keywords:** meditation, amygdala reactivity, emotional processing, anxiety, well-being

## Abstract

While meditation has drawn much attention in cognitive neuroscience, the neural mechanisms underlying its emotional processing remains elusive. Sant Mat meditators were recruited, who adopt a loving-kindness mode of meditation along with a vegetarian diet and an alcohol-restricted lifestyle and novices. We assessed their State-Trait Anxiety Inventory (STAI) and scanned their amygdala reactivity in response to an explicit and implicit (backward masked) perception of fearful and happy faces. In contrast with novices, meditators reported lower STAI scores. Meditators showed stronger amygdala reactivity to explicit happiness than to fear, whereas novices exhibited the opposite pattern. The amygdala reactivity was reduced in meditators regardless of implicit fear or happiness. Those who had more lifetime practice in meditation reported lower STAI and showed a weaker amygdala response to fear. Furthermore, the amygdala in meditators, relative to novices, had a stronger positive functional connectivity with the ventrolateral prefrontal cortex (PFC) to explicit happiness, but a more negative connectivity with the insula and medial orbitofrontal cortex (OFC) to explicit fear. Mediation analysis indicated the amygdala reactivity as the mediator for the linkage between meditation experience and trait anxiety. The findings demonstrate the neural correlates that underpin the beneficial effects of meditation in Sant Mat. Long-term meditation could be functionally coupled with the amygdala reactivity to explicit and implicit emotional processing, which would help reduce anxiety and potentially enhance well-being.

## Introduction

Meditation helps relieve anxiety and enhance well-being (Goyal et al., 2014). Mindfulness-based meditation has drawn enormous attention in the research field and is increasingly employed as a potential alternative treatment for mental illness. This type of meditation encompasses a broad array of complex practices and exhibits large variations in the way that mindfulness is conceptualized and practiced (Chiesa and Malinowski, 2011; Chiesa, 2013). Among all, the most widely studied forms of meditation include focused-attention meditation (FAM), open-monitoring meditation (OMM) and loving-kindness meditation (LKM; Hofmann et al., 2011; Lee et al., 2012; Lippelt et al., 2014; Tang et al., 2015). FAM practitioners focus their entire attention on an object or a bodily sensation with the goal to achieve a clear (vivid) and unwavering (calm and stable) state free from distraction. As for OMM, the focus of meditation becomes the monitoring of awareness itself (Vago and Silbersweig, 2012). In contrast to FAM, there is no longer an object or event in the internal or external environment for the OMM meditators to focus on. Instead, the goal of OMM is rather to stay in the monitoring state, remaining attentive to any experience that might arise, without selecting, judging or focusing on any specific object. LKM incorporates the elements of both FAM and OMM, but with an additional emphasis on orienting the practitioner towards enhancing unconditional, universal love, and positive emotional states of kindness and compassion (Lee et al., 2012). Sant Mat practitioners adopt an LKM lifestyle and FAM practices. Meaning the “Path of the Saints,” Sant Mat reflects the core teachings of saints and mystics through the ages and includes the five following precepts: (1) ahimsa or nonviolence; (2) truthfulness and avoidance of sexual misconduct; (3) love for all regardless of caste, creed, wealth or intellectual attainments; (4) the maintenance of a strict vegetarian diet; and (5) avoidance of drugs and alcohol.

Meditation enables anxiety relief and stress reduction (Marchand, 2014; Boccia et al., 2015). Brain alterations, independent of a specific style or practice, that might constitute part of the underlying neural correlates of long-term meditation include gray matter volume, white matter integrity, connectivity, cortical thickness and gyrifications (Luders et al., 2009, 2011, 2012a,b; Fox et al., 2014, 2016; Luders, 2014). Participants, who were involved in Buddhist traditions with an LKM lifestyle and FAM practices, yielded beneficial effects concerning the reduction of anxiety-related insular and amygdalar activities during anticipation and the experience of painful stimuli (Lutz et al., 2013).

Not surprisingly, previous neuroimaging studies suggest that LKM could enhance activation of brain areas that are involved in explicit emotional processing (Hofmann et al., 2011). In real life, emotion comprises a complex, continuous interplay of explicit and implicit processes. The latter appears fast, uncontrolled and automatic, independent of subjective awareness (Pessoa and Adolphs, 2010; Tamietto and de Gelder, 2010). The amygdala reactivity in response to implicit threatening (fearful and angry) faces predicted individual variability in anxiety levels (Bishop et al., 2004; Etkin et al., 2004; Pessoa et al., 2006). While previous findings indicate neural mechanisms regarding symptom improvements in generalized anxiety disorder during explicit emotion processing, how meditation modulates implicit emotional processing remains unclear (Hölzel et al., 2013).

Moreover, how practicing meditation alters low-level affective arousal through the neural mechanisms implicated in explicit and implicit emotional regulation, has also remained elusive. The former demands conscious effort for the initiation and active monitoring of emotional states in order to implement their reappraisal, whereas the latter led to completion without any conscious monitoring or inhibition of fear and conflict (Etkin et al., 2015). The beneficial effects of meditation comprise of: an improvement of stress-related health outcomes (e.g., anxiety relief) by reducing emotional interference of unpleasant stimuli (Ortner et al., 2007), facilitated emotional resilience in response to stress (Goleman and Schwartz, 1976) and emotional valence discrimination (Nielsen and Kaszniak, 2006; Robins et al., 2012). Specifically, mindfulness meditation helped direct attention towards the transitory nature of momentary experiences to regulate negative emotions (Farb et al., 2013). Compassion cultivation training adjusted to a positive orientation towards suffering (Jazaieri et al., 2014). Notably, amygdala reactivity and functional connectivity reflect one of the neuroimaging manifestations of emotion regulation (Wager et al., 2008; Lutz et al., 2014). In addition, explicitly and implicitly perceived emotionality (fear and anger) exhibited a dissociated pattern of amygdala activity, which meditated emotional mismatch negativity, an automatic electrophysiological index of emotional voice salience (Chen et al., 2014, 2015, 2016a,b), on predicting individual level of trait anxiety (Chen et al., 2017).

In this study, we used the established paradigm to clarify how long-term meditation alters amygdala activity in explicit and implicit emotional processing and whether this modulation depends on a positive or negative valence of emotion (fear or happiness). We recruited experienced meditators in Sant Mat and an fMRI in conjunction with a backward masking paradigm, was performed on them. Self-reported anxiety was measured to index the beneficial effect of long-term meditation. Moreover, we subsequently assessed functional connectivity to examine how long-term meditation modulates amygdala activity through the integration of different brain systems. Furthermore, statistical mediation analyses were conducted to identify brain mediators of anxiety change, caused by meditation experience.

## Materials and Methods

### Subjects

Twenty-one experienced meditators (14 females) and 20 novices (12 females) participated in the study after providing written informed consent and received monetary compensation. The participants were matched in terms of sex (*X*^2^ = 0.404, *p* = 0.525), age (*M* ± *SD* = 52.4 ± 7.8 vs. 50.0 ± 8.2 years; *t* = 0.964, *p* = 0.341) and education levels (*X*^2^ = 2.533, *p* = 0.639). The meditators were recruited from the Sant Mat meditation association. Each meditator regularly practiced more than 4 h per day and had sustained their practice for longer than 4 years (range = 4–26 years, *M* = 13.0, SD = 6.1). In addition to abstaining from alcohol and maintaining a strict vegetarian diet, Sant Mat practitioners enacted unconditional compassion along with the core emphasis of LKM. The novices, who had no experience with meditation, were healthy volunteers from the local community. All participants were prescreened to exclude comorbid neuropsychiatric disorders (e.g., dementia and seizures), history of head injury and alcohol or substance abuse or dependence within the past 5 years. The participants had normal or corrected-to-normal vision at the time of testing. This study was approved by the Ethics Committee of the National Yang-Ming University and conducted in accordance with the Declaration of Helsinki.

### Stimuli

The visual stimuli for fMRI scanning consisted of the black and white pictures of male and female faces showing happy, fearful and neutral facial expressions, which were chosen from the Pictures of Facial Affect (Ekman and Friesen, 1976). The faces were then cropped into an elliptical shape, the background, hair and jewelry cues of such faces were eliminated, and the faces themselves were oriented to maximize inter-stimulus alignment of the eyes and mouths. Finally, the faces were artificially colorized (red, yellow, or blue) and equalized for luminosity.

### Procedures

Before fMRI scanning, participants were required to fill in the State-Trait Anxiety Inventory (STAI) to determine their self-reported anxiety levels (Spielberger et al., 1970). The STAI consists of two 20-item scales, one for state anxiety (STAI-S) and one for trait anxiety (STAI-T). Each item is scored on a 4-point Likert-type scale with a total score range of 20–80. STAI-S verifies anxiety in specific situations, it is defined as a temporary, ever-changing emotional state by subjective, explicitly perceived feelings of apprehension and tension. STAI-T determines anxiety as a general trait, and refers to the stable tendency to attend to, experience, and report negative emotions such as fears, worries and anxiety across many situations. Given that the top range of the trait anxiety scale scores might suggest individuals are dealing with previously unreported anxiety disorders, we used a structural clinical interview tool to ensure that none of the participants showed evidence of this diagnosis (First et al., 1996). As expected, none of the participants were excluded based on this review.

The paradigm for fMRI scanning was derived from the work by Etkin et al. (2004). Prior to the functional run, participants were trained on the color identification task by using unrelated face stimuli in the same manner as the non-masked faces, in which they were told to focus on the faces and identify their color. Each stimulus presentation involved a 200-ms fixation cross as a cue for the subjects to focus on the center of the screen, followed by a 400-ms blank screen and a 200-ms face presentation. Participants then had 1,200-ms to respond with a key press indicating the color of the face. Non-masked stimuli consisted of a 200-ms emotional (fearful or happy) or neutral expression face; while backwardly masked stimuli consisted of a 17-ms emotional or neutral face, followed by a 183-ms neutral face mask belonging to a different individual of the same color and gender than the previous 17-ms face. Each epoch (12-s) consisted of six trials of the same stimulus type [explicit happy (CH), explicit fearful (CF), explicit neutral (CN), implicit happy (NH), implicit fearful (NF), or implicit neutral (NN)], but were randomized with respect to color and gender. The presentation order of the total 12 epochs (two for each stimulus type) and 12 fixation blocks (with a 12-s fixation cross) were pseudo-randomized (Supplementary Figure S1). To avoid stimulus order effects, we used two different counterbalanced run orders. The stimuli were presented using Matlab software (MathWorks, Inc., Sherborn, MA, USA) and were triggered by the first radio frequency pulse for the functional run. The stimuli were displayed on VisuaStim XGA LCD screen goggles (Resonance Technology, Northridge, CA, USA). The screen resolution was 800 × 600, with a refresh rate of 60 Hz. Behavioral responses were recorded by a fORP interface unit and saved in the Matlab program.

Reaction time (RT) data for each stimulus type were determined only for trials where subjects correctly identified the color of the faces (0.94 ± 0.25 s). The average accuracy (±SEM) for all stimuli was 89 ± 1%. RT difference scores were calculated by subtracting the average RT for the IN (implicit neutral) or EN (explicit neutral) trials for each subject from their corresponding IF (implicit fearful), IH (implicit happy), EF (explicit fearful), or EH (explicit happy) average RT.

Immediately after fMRI scanning, the participants underwent the detection task in which they were shown all the stimuli again and alerted to the presence of emotional (fearful or happy) faces. The subjects were administered a forced-choice test under the same presentation conditions as those during scanning and asked to indicate whether they observed an emotional face or not. The detection task was designed to assess possible awareness of the masked emotional faces. The chance level for correct answers was 33.3%. The performance was determined by calculation of a detection sensitivity index (*d*′) based on the percentage of trials in which a masked stimulus was detected when presented [“hits” (H)] and adjusted for the percentage of trials a masked stimulus was “detected” when not presented [“false alarms” (FA)]; [*d*′ = *z*-score (percentage H) − *z*-score (percentage FA), with chance performance = 0 ± 1.74]. Each participant’s detection sensitivity was separately calculated for each stimulus category and then averaged.

### fMRI Data Acquisition, Image Processing and Analysis

Functional and structural MRI data were acquired on a 3T MRI scanner (Siemens Magnetom Tim Trio, Erlanger, German) equipped with a high-resolution 32-channel head array coil. A gradient-echo, T2*-weighted echoplanar imaging (EPI) with a blood oxygen level-dependent (BOLD) contrast pulse sequence, was used for functional data. To optimize the BOLD signal in the amygdala (Morawetz et al., 2008), 29 interleaved slices were acquired along the AC−PC plane, with a 96 × 128 matrix, 19.2 × 25.6 cm^2^ field of view (FOV) and 2 × 2 × 2 mm voxel size, resulting in a total of 144 volumes for the functional run (TR = 2 s, TE = 36 ms, flip angle = 70°, slice thickness 2 mm, and no gap). A parallel imaging GRAPPA with factor 2 was used to speed up acquisition. Structural data were acquired using a magnetization-prepared rapid gradient echo sequence (TR = 2.53 s, TE = 3.03 ms, FOV = 256 × 224 mm^2^, flip angle = 7°, matrix = 224 × 256, voxel size = 1.0 × 1.0 × 1.0 mm^3^, 192 sagittal slices/slab, slice thickness = 1 mm, and no gap). Image processing and analysis were carried out using SPM8 (Wellcome Department of Imaging Neuroscience, London, UK). Whole brain activations were corrected for multiple comparisons of the family-wise error (FWE) rate at *P* < 0.05 (Supplementary Table S1).

In addition, activities in specific regions of interest (ROIs), in the case of our study from the right amygdala, were drawn according to a prior meta-analysis (22, −6, −12; Costafreda et al., 2008). ROI data were reported for significant contrast image peaks within 10 mm of these *a priori* coordinates. Using MarsBar^1^, signals across all voxels with a radius of 10 mm in the ROIs were averaged and evaluated for the masked and non-masked emotional (fearful and happy) comparisons. The individual mean parameter estimates (beta values) were then subject to mixed analysis of variance (ANOVA) to test for main effects of attention (explicit vs. implicit), emotion (fear vs. happiness), and group (meditators vs. novices) as well as group-by-attention-by-emotion interactions. Shorthand (e.g., EF−EN) was used to indicate the contrasts of regressors (e.g., explicit fearful > explicit neutral). Error bars signify SEM. To isolate the effects of the emotional content of stimuli, from other aspects of the stimuli and the task, we subtracted neutral (EN) or masked neutral (IN) activity from emotional (EF and EH) or masked emotional (fearful and happy) activity (IF and IH), respectively. The explicit perception of fearful and happy faces was denoted as non-masked fear and happiness (EF−EN and EH−EN) and the implicit perception of fearful and happy faces was denoted as masked fear and happiness (IF−IN and IH−IN). Voxel-wise activations were reported at *P* < 0.05 and corrected for multiple comparisons across the whole volume to control the FWE rate at *P* < 0.05.

### Functional Connectivity Analysis

The psychophysiological interaction (PPI), assesses the hypothesis that the activity in one brain region can be explained by an interaction between cognitive process and hemodynamic activity in another brain region. The interaction between the first and second regressors represented the third regressor. The individual time series for the right amygdala was obtained by extracting the first principle component from all raw voxel time series in a sphere (3 mm radius) centered on the coordinates of the subject-specific amygdala activations. These time series were mean-corrected and high-pass filtered to remove low-frequency signal drifts (128-s cut-off). The physiological factor was then multiplied by the psychological factor to constitute the interaction term. PPI analyses were then carried out for each subject involving the creation of a design matrix with the interaction term, the psychological factor and the physiological factor as regressors. PPI analyses were separately conducted for each group (meditators vs. novices), in order to identify brain regions showing significant changes in functional coupling with the amygdala, during explicitly perceived emotions in relation to meditation. Subject-specific contrast images were then entered into random effects analyses to compare the group effect. Monte Carlo simulation implemented using AlphaSim (Ward, 2000), determined that a 5-voxel extent at a height threshold of *p* < 0.005 uncorrected, yielded a FWE corrected threshold of *p* < 0.05, accounting for spatial correlations in neighboring voxels.

### Mediation Analysis

Given that a mediation analyses can be used to investigate the role of intermediate variables that lie on the causal path between two variables, it provides a decent way to identify the brain mediators of behavioral changes, by applying data from functional neuroimaging (Atlas et al., 2010; Lindquist, 2012). Here, we conducted a mediation analyses to examine the causal relationship between meditation experience in years, emotional processing in the amygdala and the reduction of anxiety.

Path *a* coded the link in which the predictor variable must be related to the mediator. Path *b* coded the link in which the mediator must be directly related to the outcome, controlling the predictor variable. The mediation effect (a*b) must be significant, which amounts to a statistical test on the product of the *a* and *b* path coefficients. Equivalently, the test for the predictor-outcome relationship would be significantly reduced by the inclusion of the mediator in the path model. We refer to the overall predictor-outcome relationship as the *c* effect and control the direct effect for the mediator as *c*’. The a*b effect was to test the significance of *c—c*’ (Supplementary Figure S2).

## Results

### Behavioral Performance

**Figure 1:**
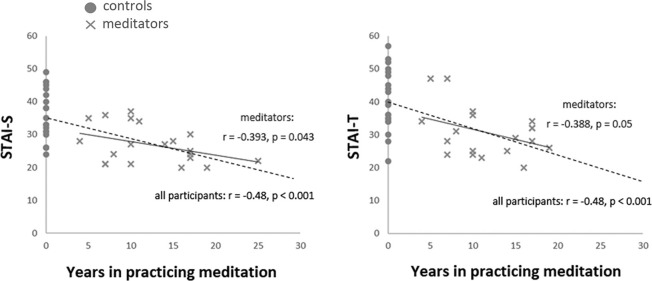
Correlation between the years of meditation experience and anxiety. The years of meditation experience were negatively correlated with the scores of State-Trait Anxiety Inventory (STAI) across all participants (STAI-S: *r*_(41)_ = −0.48, *p* = 0.001; STAI-T: *r*_(41)_ = −0.48, *p* = 0.001, dash line) and among meditators (STAI-S: *r*_(20)_ = −0.393, *p* = 0.043, one-tailed; STAI-T: *r*_(20)_ = −0.388, *p* = 0.05, one-tailed, solid line).

The accumulated life-time practice of meditation ranged from 4 to 26 years (mean ± SD: 12.95 ± 6.1) in the meditator group. The length of practice was not correlated with age (*r* = −0.069, *P* > 0.05). The STAI assessments indicated that the meditator and novice groups significantly differed in state anxiety (STAI-S; *t*_(39)_ = −3.87, *p* < 0.001; meditators (mean ± SE): 27.38 ± 1.32; novices: 35.75 ± 1.73; mean differences: −8.37 ± 2.17; 95% confidence interval: −12.75 to −3.99) and trait anxiety (STAI-T; *t*_(39)_ = −3.72, *p* = 0.001; meditators: 30.95 ± 1.55; novices: 40.5 ± 2.06; mean differences: −9.55 ± 2.57; 95% confidence interval: −14.74 to −4.36). Compared to the novice group, the meditator group exhibited lower scores overall in the STAI, regardless of state or trait anxiety. The years of meditation experience were negatively correlated with the scores of state (STAI-S; *r*_(41)_ = −0.48, *p* = 0.001) and trait anxiety (STAI-T; *r*_(41)_ = −0.48, *p* = 0.001; Figure 1 and Table 1).

**Table 1:** Demographic and descriptive statistics of each group.

	Sant Mat		Controls	
	*n* = 21		*n* = 20	
	7 males		8 males	
	Mean	SD	Mean	SD
Age (years)	52.43	7.85	49.7	8.2
Gender (male %)	33.33		40
Life-time practice of meditation (years)	12.95	6.1	NA	
STAI-T	31**	7.1	40.5	9.2
STAI-S	27.4***	6.1	35.8	7.7
RT (color identification)				
Implicit condition (s)	0.48	0.06	0.44	0.09
Explicit condition (s)	0.49*	0.08	0.43	0.09
ACC (color identification)				
Implicit condition (%)	96.3	3.3	97.2	2.6
Explicit condition (%)	95.8*	3	93.8	4.6
Hit (emotion detection)				
Implicit condition (%)	34.03	2.38	33.54	2.32
Explicit condition (%)	93.08	5.33	89.78	8.14

RT, reaction time; ACC accuracy rate; *p < 0.05; **p < 0.01; ***p < 0.001.

For the color identification task within the fMRI scanning, the data from one meditator was missing due to equipment problems. Two (group: meditators vs. novices) × two (attention: explicit vs. implicit) × three (emotion: fear vs. happiness vs. neutral) ANOVA on the RT yielded a main effect of the group (*F*_(1,39)_ = 4.54, *p* = 0.039, *η*^2^ = 0.104) and an interaction of attention by emotion (*F*_(1,39)_ = 7.84, *p* = 0.001, *η*^2^ = 0.167). Compared with novices, the meditators showed longer RTs on the color identification task, regardless of each meditators attention or emotion (meditators: 0.48 ± 0.17 s; novices: 0.43 ± 0.17 s). *Post hoc* analyses indicated that the emotion effect existed only for the implicit condition (*F*_(2,78)_ = 8.35, *p* = 0.001, *η*^2^ = 0.176, happy: 0.47 ± 0.01, fearful: 0.44 ± 0.01, neutral: 0.45 ± 0.01), rather than the explicit condition (*F*_(2,78)_ = 1.55, *p* = 0.22, η^2^ = 0.038, happy: 0.45 ± 0.01, fearful: 0.47 ± 0.01, neutral: 0.46 ± 0.01). Furthermore, within each condition [explicit happy (EH), explicit fearful (EF), implicit happy (IH), and implicit fearful (IF)], the RT was not correlated with the age within each group (all *p* > 0.2; Table 1).

For the accuracy rate of color identification, ANOVA did not exhibit any effect except a main effect of attention (*F*_(1,39)_ = 12.3, *p* = 0.001, η^2^ = 0.24) and an interaction of the group by attention (*F*_(1,39)_ = 6.33, *p* = 0.016, η^2^ = 0.14). The main effect of emotion (*F*_(2,78)_ = 0.05, *p* = 0.95), group (*F*_(1,39)_ = 0.38, *p* = 0.54), and other interactions (all *p* > 0.1) was not significant. A *post hoc* analyses indicated that while the accuracy rate of color identification was overall higher in the implicit condition (explicit: 96.7 ± 0.5; implicit: 94.8 ± 0.6), the attention effect existed only for the novices (explicit: 93.8 ± 0.8; implicit: 97.2 ± 0.6) rather than the meditators (explicit: 95.8 ± 0.9; implicit: 96.3 ± 0.7; Table 1).

For the detection task outside the fMRI scanner, according to a one-tailed binominal model, scores of 28 hits (42.2% hit rate) and above were considered a significant over chance level (33%). Both the meditators and novices performed above chance in the explicit condition (*d*′, mean ± SD: 2.63 ± 0.57; 2.31 ± 0.62), and below chance in the implicit condition (0.04 ± 0.06; 0.03 ± 0.07). Between the groups, the hits were not significantly different (explicit condition: *t*_(39)_ = 1.54, *p* = 0.13; meditators: 0.93 ± 0.05; novices: 0.90 ± 0.08; implicit condition: *t*_(39)_ = 0.69, *p* = 0.5; meditators: 0.34 ± 0.02; novices: 0.34 ± 0.02; Table 1).

### fMRI Data

The voxel-wise analysis identified the hemodynamic changes between the groups in response to explicit and implicit emotional processing (Tables 2, 3; Supplementary Table S1). In response to explicit happiness [EH−EN], greater signal changes in the right amygdala and superior temporal gyrus were observed in the meditators, when compared to the novices [(EH−EN)|meditators > (EH−EN)| novices]. The reversed comparison [(EH−EN)| novices > (EH−EN)|meditators] showed an increased activity in the thalamus and the anterior cingulate cortex. In response to explicit fear (EF−EN), as compared with the novices, the meditators showed a decreased signal in the bilateral amygdala, thalamus/midbrain, medial orbitofrontal cortex (OFC), right lingual gyrus, and the superior temporal gyrus [(EF−EN)|meditators < (EF−EN)| novices]. The reversed comparison [(EF−EN)|meditators > (EF−EN)| novices] did not yield significant results. For implicit happiness processing (IH−IN), as compared with the novices, the meditators showed a decreased signal change in the right amygdala, precuneus, left lingual gyrus, and the parahippocampus [(IH−IN)|meditators < (IH−IN)| novices]. The reversed comparison [(IH−IN)|meditators > (IH−IN)| novices] showed an increased activity in the cuneus. In response to implicit fear (IF−IN), as compared with the novices, the meditators showed a decreased signal change in the right amygdala, medial frontal gyrus, left hippocampus, thalamus, and the parahippocampus [(IF−IN)|meditators < (IF−IN)| novices]. The reversed comparison [(IF−IN)|meditators > (IF−IN)| novices] did not yield significant results.

**Table 2:** Group-wise fMRI results to explicit emotional processing.

		MNI coordinates				
Brain regions	Side	*x*	*y*	*z*	*t*-value	*k*
**Explicit happy (EH) vs. Neutral (EN)**						
**Meditators > Novices**						
Superior temporal gyrus	R	40	14	−18	3.76	10
Amygdala	R	22	0	−16	2.73*	13
**Novices > Meditators**						
Thalamus/Midbrain	L	−16	−22	−4	3.2	5
Anterior cingulate cortex	R	6	34	0	3.19	10
**Explicit fearful (EF) vs. Neutral (EN)**						
**Meditators > Novices**						
NS						
**Novices > Meditators**						
Amygdala	L	−24	−6	−14	1.97*	6
Amygdala/Parahippocampus	R	26	−12	−8	4.11	20
Thalamus/Midbrain	R	8	−28	−8	3.4	5
Lingual gyrus	R	22	−56	−6	4.32	8
Superior temporal gyrus	R	40	−42	8	3.98	12

*All clusters are significant at FWE-corrected P < 0.05 [thresholded at P < 0.001, cut-off, and t = 3.145 (uncorrected) with a spatial extent threshold k > 5], except those marked with an asterisk, which are taken from predefined regions of interest (ROIs) and significant at uncorrected P < 0.05*.

**Table 3:** Group-wise fMRI results to implicit emotional processing.

		MNI coordinates				
Brain region	Side	*x*	*y*	*z*	*t*-value	*k*
**Implicit happy (IH) vs. Neutral (IN)**						
**Meditators > Novices**						
Cuneus	L	−14	−70	16	3.66	7
**Novices > Meditators**						
Amygdala	R	20	4	−10	2.38*	13
Lingual gyrus	L	−28	−44	−2	4.02	7
Precuneus	R	20	−52	−4	3.95	5
Parahippocampus	L	−18	−46	0	3.81	7
**Implicit fearful (IF) vs. Neutral (IN)**						
**Meditators > Novices**						
NS						
**Novices > Meditators**						
Amygdala	R	18	−4	−16	3.02*	14
medial PFC	R	18	30	−6	3.71	8
Hippocampus	L	−32	−40	0	3.95	8
Thalamus	L	−22	−24	0	3.75	7
Parahippocampus	L	−18	−46	−2	4.98	13

*All clusters are significant at FWE-corrected P < 0.05 [thresholded at P < 0.001, cut-off, and t = 3.143 (uncorrected) with a spatial extent threshold k > 5], except those marked with an asterisk, which are taken from predefined ROIs and significant at uncorrected P < 0.05. Abbreviations: medial PFC, medial prefrontal cortex*.

**Figure 2:**
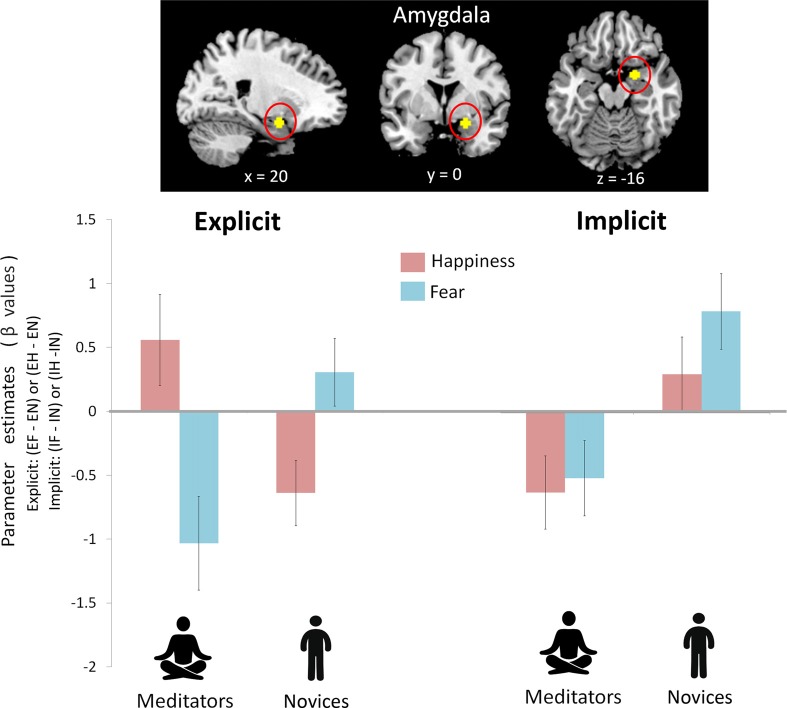
Amygdala reactivity between mediators and novices during explicit and implicit emotional processing. A prior region of interest (ROI)-based beta estimates were extracted from the amygdala. There was an interaction of group (mediators vs. novices) × attention (explicit vs. implicit) × emotion (happiness vs. fear; *F*_(1,39)_ = 4.91, *p* = 0.033). *Post hoc* analysis indicated a group × emotion interaction during the explicit condition (*p* = 0.001) and a group effect during the implicit condition (*p* < 0.001). During the explicit condition, to perceived happiness compared with fear (EH−EF), amygdala reactivity was increased in the meditators but was decreased in the novices. During the implicit condition, compared with the novices, the meditators showed significantly weaker amygdala reactivity regardless of fear or happiness.

To avoid circular inferences, prior ROI-based beta estimates were extracted from the amygdala (Figure 2). ANOVA analysis with two within-subject variables (attention: explicit vs. implicit; and emotion: fear vs. happiness) and one between-subject variable (group: meditators vs. novices) revealed an interaction of group × attention × emotion (*F*_(1,39)_ = 4.91, *p* = 0.033, η^2^ = 0.11). A *post hoc* analyses indicated an interaction of group × emotion only under the explicit condition (*F*_(1,39)_ = 13.71, *p* = 0.001, η^2^ = 0.26), but not under the implicit condition (*F*_(1,39)_ = 0.33, *p* = 0.57, η^2^ = 0.008). The follow-up analyses indicated that the emotion effect in the amygdala had opposite directions depending on the factor of group. When explicitly perceived happiness (EH−EN) was compared with fear (EF−EN), amygdala reactivity was increased in the meditators (*F*_(1,20)_ = 4.10, *p* = 0.05, η^2^ = 0.17; happiness: 0.56 ± 0.36; fear: −1.03 ± 0.26) but was decreased in the novices (*F*_(1,19)_ = 6.46, *p* = 0.02, η^2^ = 0.25; happiness: −0.64 ± 0.37; fear: 0.31 ± 0.26). On the other hand, a main effect of the group was shown under the implicit condition (*F*_(1,39)_ = 20.69, *p* < 0.001, η^2^ = 0.35). Compared with novices, meditators showed significantly weaker amygdala reactivity regardless of fearful (IF−IN, meditators: −0.51 ± 0.29; novices: 0.79 ± 0.3) or happy (IH−IN, meditators: −0.62 ± 0.29; novices: 0.3 ± 0.29) processing.

Correlation analyses showed that higher trait (STAI-T) and state (STAI-S) anxiety was associated with increased right amygdala activity during explicit fear (EF−EN) (STAI-T: *r*_(41)_ = 0.41, *p* = 0.007; STAI-S: *r*_(41)_ = 0.39, *p* = 0.012), as well as a trend approaching significance during implicit fear (IF−IN) (STAI-T: *r*_(41)_ = 0.29, *p* = 0.06; STAI-S: *r*_(41)_ = 0.27, *p* = 0.09). Furthermore, across the whole sample, the right amygdala reactivity was negatively modulated by the amount of meditation experience [explicit fear (EF−EN): *r*_(41)_ = −0.44, *p* = 0.04; implicit fear (IF−IN): *r*_(41)_ = −0.4, *p* = 0.01). Participants who had more lifetime practice in meditation showed less amygdala response during fear processing. The relationship between meditation experience and amygdala reactivity was significant after adjusting for age, sex and measurement error (explicit: *β* = −0.42, *P* = 0.007; implicit: *β* = −0.38, *P* = 0.016).

### Functional Connectivity

**Figure 3:**
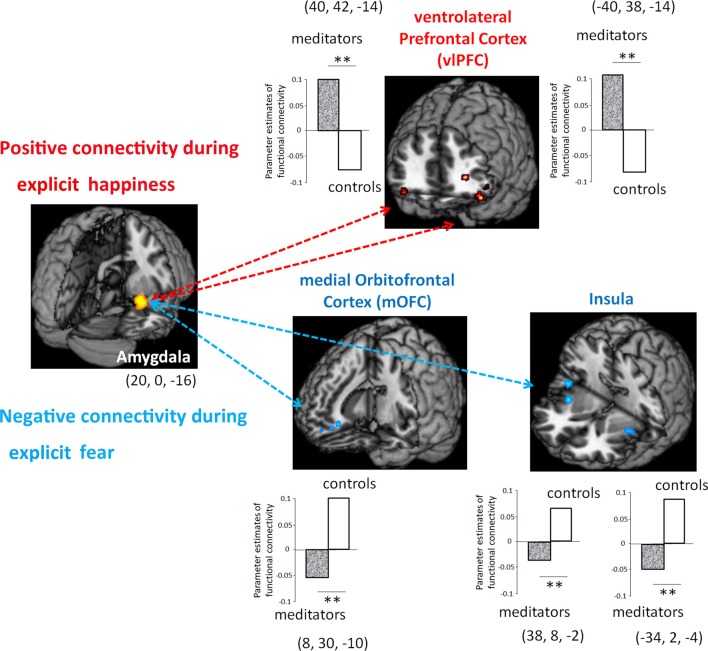
Functional connectivity between meditators and novices during explicit emotional processing. Compared with the novices, the meditators had a significantly more positive connectivity of the amygdala with the ventrolateral prefrontal cortex (PFC) during the processing of happiness, whereas a significantly more negative connectivity of the amygdala with the medial orbitofrontal cortex (OFC) and the bilateral insula during the processing of fear. **P < 0.01.

To further examine to which extent meditation-induced modulation in low-level affective processing of the amygdala contributed to the functional coupling between different brain regions, we subsequently assessed functional connectivity in the area (i.e., amygdala) where functions were related to meditation (Figure 3). To estimate how meditation altered the functional connectivity of the amygdala in response to explicit emotional processing as manifested by the voxel-wise analysis results, the time series of the first eigenvariates of the BOLD signal were temporally filtered, mean corrected, and deconvolved to generate the time series of the neuronal signal for the source region—the right amygdala (22, 0, −16)—as the physiological variable in PPI analysis. Selected as the PPI source region, the physiological regressor was denoted by the activity in the right amygdala. Emotion (fear: EF−EN and happiness: EH−EN) was the psychological regressor. During explicit emotional processing, as compared with the novices, the meditators showed a significantly stronger positive coupling with the right and left ventrolateral prefrontal cortex (PFC; 40, 42, −14; −40, 38, −14) for happiness. For fear, compared to the novices, the meditators showed a significantly more negative connectivity between the amygdala and medial OFC (8, 30, −10) as well as between the amygdala and bilateral insula (right: 38, 8, −2 and left: −34, 2, −4). Novices did not show any connectivity that exceeded that of the meditators.

### Mediation Analysis

The Mediation Effect Parametric Mapping was used to test a specific hypothesis about brain-behavior relationships (Baron and Kenny, 1986; Wager et al., 2008). Based on the conceptual framework of a mediation effect (MacKinnon et al., 2007) and given the association between the amount of meditation experience and anxiety (STAI), the years of meditation experience were selected as the predictor, the STAI scores as the outcome, and the hemodynamic activity in the right amygdala as the mediator.

**Figure 4:**
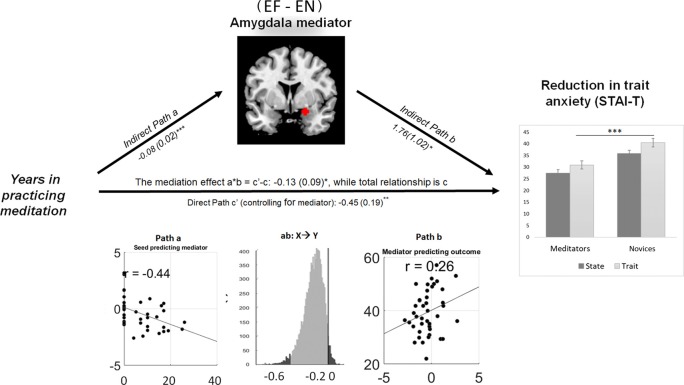
Amygdala reactivity to explicit fear mediating the meditation effect on the reduction of trait anxiety. Path diagram demonstrates the causal relationship between years of meditation experience (left panel), right amygdala (22, 0, −16) activity (top) and trait anxiety (right panel) in the path model. Accumulated lifetime practice (left) as the predictor variable predicts the hemodynamic activity in the amygdala (top). The connection of meditation experience to the brain mediator (amygdala) is the “*a*” path. The lines are labeled with path coefficients, and standard errors are shown in parentheses. The connection of the amygdala mediator to the outcome (trait anxiety) is the “*b*” path. They are calculated controlling for meditation experience and for the amygdala mediator, as the standard in mediation models. The direct path is the *c*’ path, which is calculated controlling for brain mediator. Bottom panel visualizes the path *a*, *b* and the mediation effect of a*b. ***p < 0.001, **p < 0.01, *p < 0.05, one-tailed.

The right amygdala mediated the linkage between meditation experience and trait anxiety. During explicit fear processing (EF−EN), the hemodynamic activity in the amygdala was negatively modulated by the years of meditation experience and predicted the reduction of trait anxiety in the same direction: *a* = −0.08, *Z* = 3.65, *p* < 0.001; *b* = 1.76, *Z* = 1.74, *p* = 0.03; and a*b = −0.13, *Z* = −1.73, *p* = 0.04 (Figure 4).

## Discussion

Behaviorally, we found that Sant Mat meditators, who adopted a loving-kindness stance in meditation along with a vegetarian diet and alcohol-restricted lifestyle, significantly reported lower scores overall on the STAI, regardless of state or trait anxiety. Those who had more lifetime practice in meditation reported lower anxiety and showed less amygdala response to perceived fear. Furthermore, the novices showed increased hypervigilance as demonstrated by their performance in the color identification task, where their RTs where seen to be shorter than that of the meditators. Shorter RTs have been previously linked to higher trait anxiety when utilizing a similar task (Etkin et al., 2004). Thus, this reduction in hypervigilance in the meditators, could be a manifestation of the beneficial effects in anxiety reduction resulting from Sant Mat Similarly, while the explicit perception of emotion generally hampered the accuracy performance of color identification, compared to the effect caused by implicit emotional processing, meditators remained less affected by and less hypervigilant toward explicit emotions. In the same vein, practicing FAM caused a beneficial effect on self-reported anxiety in patients with general anxiety disorder (Hoge et al., 2013a). Individuals previously reported lower STAI scores after a course of meditation training (Coppola, 2007). Cross-sectional studies have revealed that both long-term FAM and LKM induce alterations in cognitive abilities, brain activity, connectivity and even neurotransmitters (Lutz et al., 2004; Brefczynski-Lewis et al., 2007; Luders et al., 2009; Lykins and Baer, 2009; Jacobs et al., 2011; Hoge et al., 2013b; Luders, 2014). One longitudinal study that experimentally extended cross-sectional findings in FAM practitioners, indicated that changes in resting-state functional connectivity explained 30% of the overall meditation training effects on reducing interleukin-6, a biomarker of stress-related systemic inflammation (Creswell et al., 2016).

Although there are several neuroimaging studies that have probed emotion regulation associated with meditation, they typically present study participants with affective pictures (Taylor et al., 2011; Allen et al., 2012; Desbordes et al., 2012; Hölzel et al., 2013; Lutz et al., 2014), words and/or statements (Farb et al., 2007; Goldin et al., 2013) for them to explicitly evaluate. But how meditation modulates implicit emotional processing and its relation to anxiety has not been addressed yet. Based on the established paradigm for emotional processing, we found an interaction of the group (meditators vs. novices) × attention (explicit vs. implicit) × emotion (happiness vs. fear) in amygdala reactivity. A *post hoc* analyses indicated that there was an interaction between a group and an emotion under the explicit condition but a group effect under the implicit condition. Interestingly, the meditators showed stronger amygdala reactivity to explicit happiness than to fear, whereas the novices exhibited the opposite effect. In contrast to the novices, the meditators showed significantly weaker amygdala reactivity regardless of implicit fearful or happy processing. A mediation analyses showed that meditation-induced anxiety reduction was mediated by the amygdala reactivity. During explicit fear processing, the hemodynamic activity in the amygdala was negatively modulated by the years of meditation experience and predicted the reduction of trait anxiety in the same direction. Furthermore, meditators, compared with novices, exhibited a positive functional connectivity between amygdala and ventrolateral PFC in response to explicit happiness, but a negative connectivity between amygdala, insula and medial OFC in response to explicit fear. These findings could be ascribed to emotional regulation where implicit low-order information was processed and coalesced with other kinds of neural signals in the cognitive assembly of explicit emotional experiences, by the recruitment of cortical and subcortical circuits to support subjective feelings (Wager et al., 2008; Lutz et al., 2014; LeDoux and Brown, 2017). As FAM and LKM activated distinct brain circuits involved in emotion regulation (Salzberg, 1995; Lee et al., 2012; Garrison et al., 2014; Tang et al., 2016; Guendelman et al., 2017) and the present findings from Sant Mat appear parallel to their combined effect.

In response to implicit (masked) emotional processing, the meditators showed reduced amygdala reactivity compared to the novices, regardless of fear or happiness, suggesting an arousal reduction to emotional stimuli. This result could offer evidence to support the notion that implicit emotion regulation underpins the beneficial effects of meditation (Gross, 2014). Moreover, amygdala activation to emotionally neutral stimuli was seen to be elevated in individuals who were carriers of the risk allele of the serotonin transporter polymorphism (Canli and Lesch, 2007). The aforementioned findings suggest that such individuals are hypervigilant even in regard to emotionally neutral stimuli in the environment, thus, regarding these stimuli as more anxiogenic than non-carriers of this risk allele do. Another study found that stronger amygdala activation to neutral stimuli was coupled with higher levels of trait anxiety, specifically during implicit, but not explicit, processing (Chen et al., 2017). Furthermore, fearful faces *per se* as well as their interaction with mask types (faces vs. patterns) were supposed to modulate amygdala activity. When fearful faces were masked by neutral faces, amygdala activity was increased. By contrast, when fearful faces were masked by non-face patterns, amygdala activity was decreased (Kim et al., 2010). In this study, and surprisingly enough, Sant Mat meditators showed similar amygdala deactivation during implicit processing as did naïve participants in the pattern mask condition. Thus, it is reasonable to infer that experienced LKM meditators might exert implicit emotion regulation to reduce/inhibit affective arousal to uncertain nonconscious input.

Some limitations of this study must be acknowledged. First, although in general a stimulus that is shown for about 30-ms before masking is considered not accessible to conscious awareness, some studies applying objective criteria—instead of subjective reports—by way of signal detection methods, showed that 36% of the participants were able to detect 33-ms targets (Pessoa, 2005). This variability among participants’ “true sensitivity” to masked fearful faces appears to be linked to amygdala reactivity (Pessoa et al., 2006). While one preliminary report showed that FAM practitioners might have better access to implicit information, as manifested by better performance on the Remote Associate Test (Strick et al., 2012), we did not find any significant group difference in the hits of the detection task, regardless of the explicit or implicit condition. Future studies using objective signal detection methods to examine the accessibility of nonconsciousness in experienced meditators is warranted. A second limitation was regarding individual differences to the cross-sectional design, making the direction of causality inherently ambiguous and unable to be fully determined. The benefits of anxiety hereby highlighted, might be ascribed to the combined effects of meditation, loving-kindness spirituality and lifestyle (e.g., having a vegetarian and alcohol-restricted diet; Fox et al., 2014, 2016). Even though the association between anxiety and alcohol consumption has been demonstrated mostly among those who have a pattern of consumption that falls within the parameters of abuse or dependence for this substance (Haynes et al., 2005; Smith and Randall, 2012) and even when our participants were screened for and excluded if they had such a pattern in their history, a lower risk of anxiety for those people who abstain themselves from consuming alcohol has also been observed (Haynes et al., 2005). Thus, it is plausible to infer that the reduction of anxiety observed in Sant Mat practitioners is driven by their alcohol-restricted lifestyle. In the same manner, the type of diet (vegetarian vs. non-vegetarian) was not included as a variable in this research. Additionally, the intelligence quotient (IQ) was not obtained from any of the participants in either of the groups. These two lifestyle variables alongside the IQ, could all be possible confounders in this study, thus, future research taking these factors in to account is highly advised.

## Conclusion

Altogether, this fMRI study demonstrated the neural correlates underpinning the beneficial effects of Sant Mat on anxiety. The present findings might lend support to the higher-order theory of emotional consciousness. Long-term meditation training is functionally coupled with amygdala reactivity in response to explicit and implicit emotional processing, which in turn, is associated with reduced anxiety and a potential to enhance well-being.

## Author Contributions

CC and YC conceived and designed the experiments and wrote the first draft of the manuscript. Y-CC and K-LC collected the data. CC, Y-CC and K-LC contributed to data analysis. All authors contributed to data interpretation and manuscript write-up.

## Conflict of Interest Statement

The authors declare that the research was conducted in the absence of any commercial or financial relationships that could be construed as a potential conflict of interest.

## Footnotes

^1^http://marsbar.sourceforge.net/
