# Supplementary material for: Atypical Anxiety-Related Amygdala Reactivity and Functional Connectivity in Sant Mat Meditation
Source: Front Behav Neurosci. 2018 Dec 4;12:298. doi: 10.3389/fnbeh.2018.00298 (PMC6288484; doi:10.3389/fnbeh.2018.00298)
Supplement: Supplementary file 1 [file Data_Sheet_1.PDF]

SUPPLEMENTARY MATERIALS

Supplementary Figure 1. Design of the task.

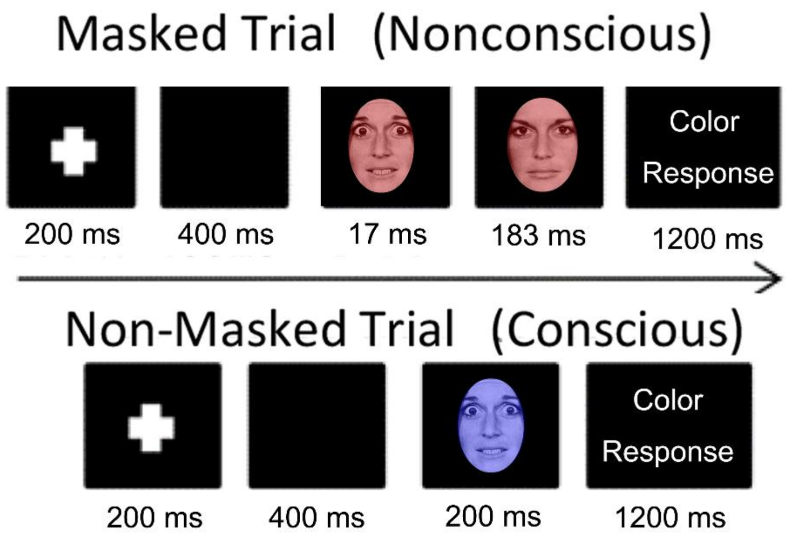

Supplementary Figure 2. Hypothesis for mediation analysis.

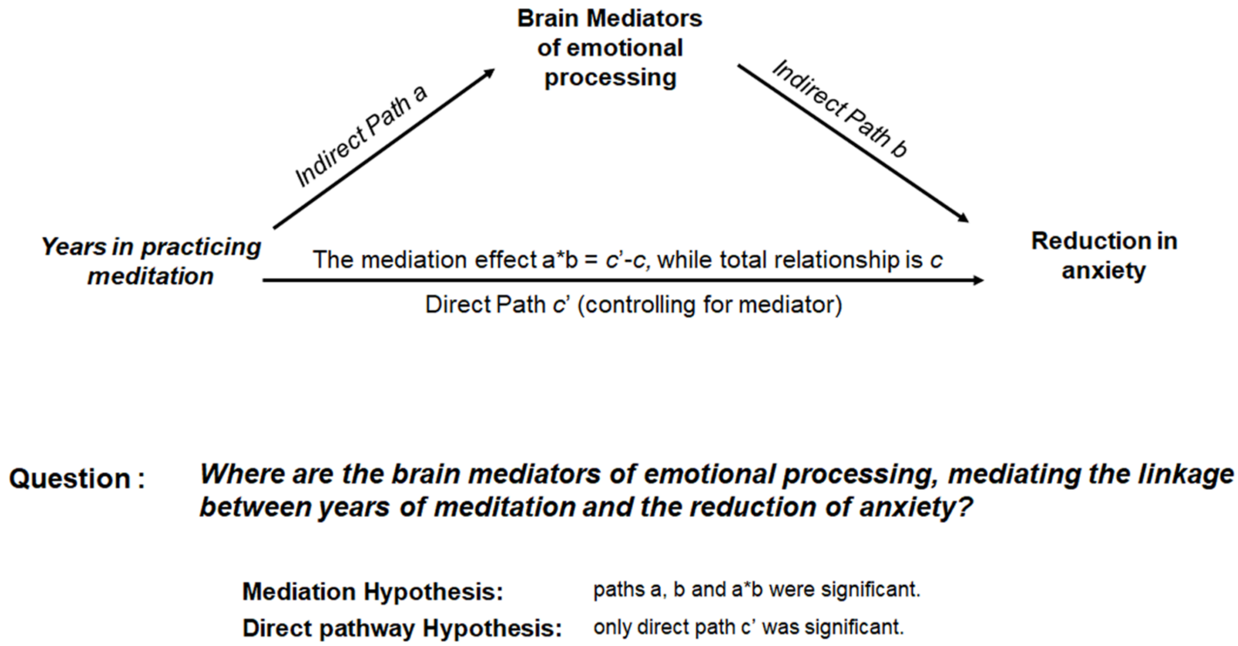

Supplementary Table 1. Pooled group results for other contrasts. All clusters are significant at FWE-corrected P < 0.05 [thresholded at P < 0.001, cut-off, and t = 3.143 (uncorrected) with a spatial extent threshold k > 5], except those marked with an

asterisk, which are taken from predefined ROIs and significant at uncorrected P < 0.05.

		MNI Coordinates				
Brain Region	side	x	y	z	*t*-value	*k*
**Nonconscious > Conscious**						
Superior occipital cortex	R	20	-88	6	4.02	15
Middle temporal gyrus	L	-56	-34	0	3.97	11
Medial orbital frontal cortex	L	-12	50	-10	3.70	6
Parahippocampa Gyrus	L	-18	-52	-8	3.69	8
Amygdala	L	-24	2	-18	2.25	*
Amygdala	R	22	4	-16	1.94	*
**Conscious > Nonconscious**						
N.S.						
**Happy > Fear**						
Orbital frontal cortex	L	-18	40	-10	4.04	10
Caudate	R	12	4	8	3.81	5
**Fear > Happy**						
Amygdala	R	-28	-4	-20	2.1	*
Parahippocampus/Amygdala	L	24	-8	-16	2.51	*
